# Direct ENIT: An easy and reliable tool for gRNA efficacy verification by tracking induced chromosomal translocation

**DOI:** 10.1016/j.mex.2020.101104

**Published:** 2020-10-16

**Authors:** Nikolai A. Lomov, Vladimir S. Viushkov, Aleksei V. Zamalutdinov, Maria D. Sboeva, Mikhail A. Rubtsov

**Affiliations:** a Department of Molecular Biology, Faculty of Biology, Lomonosov Moscow State University, Moscow, Russia; b Department of Biochemistry, Institute for Translation Medicine, I.M. Sechenov First Moscow State Medical University (Sechenov University), Moscow, Russia

**Keywords:** CRISPR/Cas, Genome editing, Guide RNA testing, Direct PCR

## Abstract

CRISPR/Cas systems (Clustered regularly interspaced palindromic repeats / CRISPR-associated) are rapidly becoming a commonplace and popular tool for gene editing in research and clinical contexts. However, the quality of CRISPR/Cas experiments depends heavily on the guide RNA (gRNA) design; therefore, a reliable, easy, and rapid method for verifying gRNA cleavage efficacy is necessary. Engineered nuclease-induced translocations (ENIT) are an easy and cost-efficient method for the verification of gRNA efficacy, which involves tracking induced chromosomal mutations, using polymerase chain reaction (PCR). We have customized this method using both direct PCR and nested PCR approaches and have been able to reduce the sample preparation time. We present a simple and reliable gRNA testing approach that requires no specific enzymes or equipment.

• The approach requires only routinely used enzymes and equipment.
• Cost- and time-efficient, requiring approximately 30 min for PCR sample preparation, without requiring DNA purification.
• High sensitivity, with induced translocation detected in 100 of 10,000 cells in the general population.

## Graphical abstract

**Figure:**
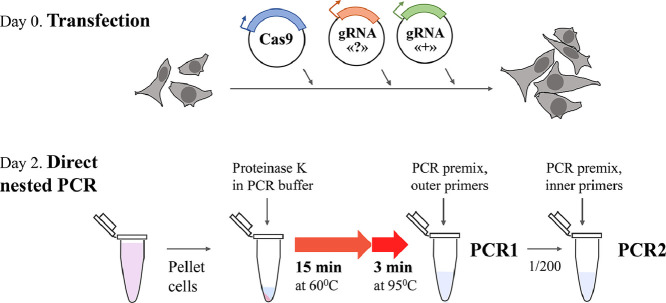

Specifications Table

**Table:** 

Subject Area	Biochemistry, Genetics and Molecular Biology
More specific subject area	Genetic engineering
Method name	Engineered Nuclease-induced Translocations (ENIT)
Name and reference of original method	D. Germini, Y. Bou Saada, T. Tsfasman, K. Osina, C. Robin, N. Lomov, M. Rubtsov, N. Sjakste, M. Lipinski, Y. Vassetzky *A One-Step PCR-Based Assay to Evaluate the Efficiency and Precision of Genomic DNA-Editing Tools,* Mol. Ther. - Methods Clin. Dev., 5 (2017), pp. 43–50. https://doi.org/10.1016/j.omtm.2017.03.001. S. Ren, M. Li, H. Cai, S. Hudgins, and P. A. Furth *A Simplified Method to Prepare PCR Template DNA for Screening of Transgenic and Knockout Mice***,***Contemp. Top. Lab. Anim. Sci.*, vol. 40, no. 2, pp. 27–30, 2001. https://pubmed.ncbi.nlm.nih.gov/11300684/
Resource availability	Availability of all reagents, plasmids, and other equipment is reported in the method details.

## Method details

**Fig. 1:**
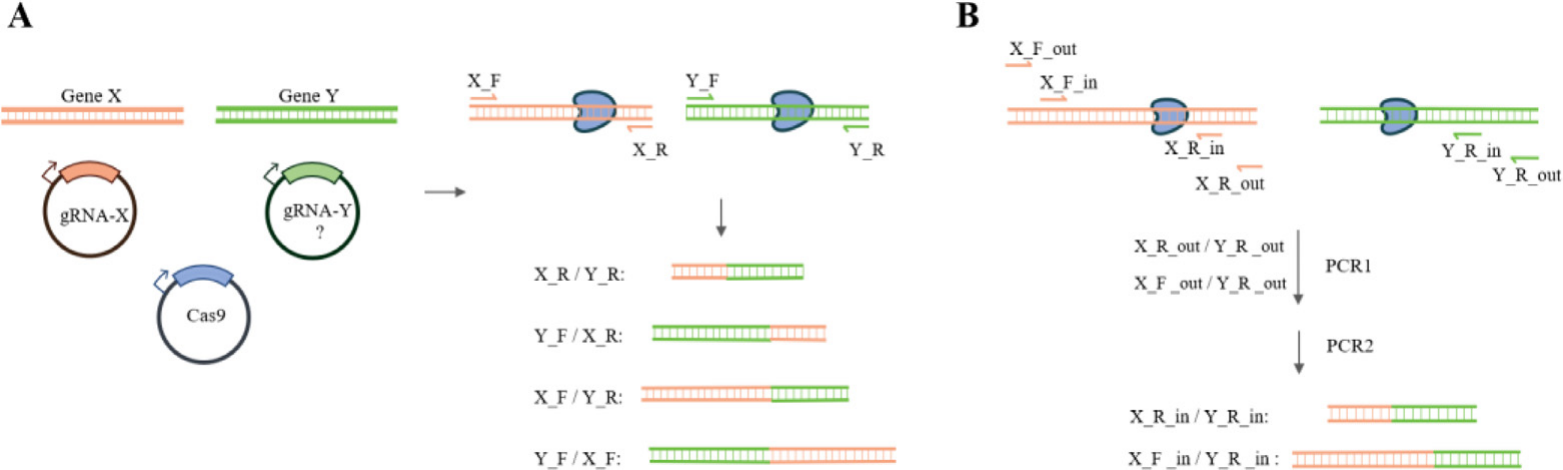
Principle of the ENIT approach. A. Cells are co-transfected with a Cas9-expressing plasmid and two plasmids encoding guide RNAs: gRNA-X, whose performance has been pre-validated, and gRNA-Y, with unknown efficacy (indicated by a question mark). The successful assembly of the CRISPR-Cas9 complex results in the induction of double-stranded breaks (DSBs) in genes X and Y, followed by the translocation of the targeted loci. Four different induced translocation variants can be detected, using the PCR primers X_F, X_R, Y_F, and Y_R, by annealing within the breakpoint junction region. B. Nested PCR approach. Primer design for the detection of only two translocation variants is shown.

The engineered nuclease-induced translocations (ENIT) approach represents one of the easiest existing methods for confirming gRNA efficacy [1]. This method relies on the detection of nuclease-induced chromosomal translocations. Cells are co-transfected with a plasmid encoding the Cas9 gene and two additional plasmids, one of which encodes a previously validated gRNA and the other encodes the new gRNA that is being tested. If the gRNA cleaving efficacy is sufficient, a chromosomal translocation will occur between the loci targeted by the two gRNAs (Fig. 1).

We have modified the standard ENIT protocol by combining it with a direct PCR approach (i.e., PCR performed without prior DNA isolation and purification). This modification makes gRNA efficacy verification both faster and less resource-consuming. Moreover, all reaction steps are performed in a single tube, preventing sample loss and increasing the overall sensitivity of gRNA efficacy testing.

In this article, we thoroughly described the Direct ENIT protocol, including primer design, cell transfection, and direct PCR, which is performed in two steps using outer and inner primer pairs (nested PCR, Fig. 1B). The full protocol was verified using HeLa cells because they are easy to cultivate and transfect; however, this protocol can likely be adapted for use in other cell lines, in both adherent and suspended cultures. We also described a series of experiments that were performed to optimize and validate this Direct ENIT protocol.

### Materials

*Cell lines*: HeLa cells were cultivated in Dulbecco's modified Eagle medium (DMEM), supplemented with 10% fetal bovine serum (FBS) and 1% penicillin-streptomycin, in an incubator with 5% CO_2_ at 37°C.

*Common equipment required for cell culture*: laminar-flow hood, humidified CO_2_ incubator, water bath, 6-well cell culture plates, 15-ml conical tubes, centrifuge, and hemocytometer or cell counter.

*Cell culture and transfection reagents*: cell culture media (DMEM and Opti-MEM^TM^, Thermo Fischer Scientific), FBS, penicillin-streptomycin solution, trypsin-EDTA solution, 1 × phosphate-buffered saline (PBS), and TurboFect^TM^ Transfection reagent (Thermo Fisher Scientific).

*Plasmids*: Cas9-expression plasmid (e.g., #41815 Addgene) and plasmids expressing gRNAs of interest (e.g., Addgene #53188).

*Common equipment for PCR*: PCR hood, 200-μl PCR tubes, thermocycler (e.g., C1000 Touch™ Thermal Cycler, Bio-Rad) and microcentrifuge (e.g., MiniSpin®, Eppendorf).

*Direct PCR reagents*: a 20 mg/ml Proteinase K solution (Thermo Fisher Scientific), DreamTaq^TM^ Hot Start DNA Polymerase (Thermo Fisher Scientific), 10 × DreamTaq^TM^ Buffer, dNTPs, and specific primers for the amplification of the translocation region.

*Agarose gel electrophoresis*: horizontal electrophoresis system, gel visualization equipment, such as a UV light box, agarose gel, DNA stain (ethidium bromide), and TAE buffer.

### Protocol

#### Primer design

Design primers for nested PCR, flanking the gRNA target sequence. Two CRISPR/Cas-mediated DSBs may result in the generation of four potential translocation variants (Fig. 1); however, the detection of a single variant is sufficient to assess gRNA efficacy. The outer primer annealing sites should be located 200-500 bp away from the CRISPR-induced breakpoint junction. The inner pair of primers should be designed to anneal closer to the breakpoint site, with an optimal resulting amplicon size of 150-500 bp, after a second amplification round. Additionally, positive control PCR primers should be utilized to detect an unrearranged locus.

#### Step 1: transfection

1. 24 h before transfection passage HeLa cells into 6-well plate. However, any preferred cell line can be used.
  NOTE: It is beneficial to change cell medium to antibiotic- and serum-free media (e.g., Opti-MEM^TM^) 3 h prior to transfection because antibiotics and sera may decrease transfection efficacy.
2. Once the cell culture reached the optimal confluency for transfection (approximately 70%–80%), co-transfect cells with Cas9 plasmid and plasmids encoding both the pre-validated and unvalidated gRNAs according to the protocol of the transfection reagent. Addition of equal amounts of each plasmid, for a total DNA quantity of approximately 3 µg per well, is recommended. We used 6 µl TurboFect^TM^ Transfection Reagent [2]; however, any preferred transfection reagent or system can be used. Non-transfected cells should be included as a negative control.

#### Step 2: direct nested PCR

1. 48 h after transfection detach the cells from a culture plate with 300 µl of 0.05% trypsin solution. Thoroughly resuspended cells in 2 ml DMEM, containing 10% FBS, to inactivate trypsin.
2. For each PCR reaction, transfer 200 µl cell suspension to a sterile PCR tube (10,000—50,000 cells). Pellet cells by centrifugation, for 2 min at 1000 rcf, and discard the supernatant.
  NOTE: Optionally, the pellet can be washed with 1 × PBS, followed by a second centrifugation of the cell suspension using the same centrifugation parameters described above.
  All subsequent sample manipulations should be performed in a PCR hood to avoid contamination.
3. Resuspend the cell pellet in 10 µl proteinase K solution (0.1 mg/ml), in 2 × PCR buffer supplied with DNA polymerase.
4. Place the samples in a thermocycler, with pre-heated lid, for 15 min at 60 °C, followed by 3 min at 95 °C.
5. To perform the first round of nested PCR, add DNA polymerase, the outer primers, dNTPs, and deionized water to the cell suspension to obtain a final volume of 20 µl. Use the reagent concentrations recommended by the DNA Polymerase manufacturer. Return the PCR tubes to the thermocycler, and perform the first round of nested PCR. The reaction conditions may vary depending on specific primer designs and amplicon sizes. We recommend using at least 1 min for primer annealing and running 20 cycles.
6. Prepare two sets of PCR tubes: one containing 156 µl deionized water and one containing 16 µl the second-round PCR mixture, including DNA Polymerase, inner primers, PCR buffer, and dNTPs. Use the reagent concentrations recommended by the DNA Polymerase manufacturer to perform a 20-µl reaction. Add a 4 µl aliquot of the first-round PCR product to the tube containing 156 µl water, mix thoroughly by pipetting the mixture repeatedly and transfer 4 µl diluted sample into the second tube containing the prepared 16 µl PCR mixture; use one filter tip for this procedure to avoid product loss and contamination. Thus, the final second-round reaction mix contains the 200-fold diluted product of the first-round PCR reaction. Thermocycling conditions should be determined based on primer design and the resulting amplicon size; however, we recommend using at least 1 min for primer annealing and running 25 cycles.
7. Analyze the second-round PCR products by agarose gel electrophoresis. The PCR reaction performed using cells without translocations should be included as a negative control. The presence of amplicons at the expected length will indicate the successful rearrangement of the targeted loci and the sufficient performance of the tested gRNA.

NOTE: Compare your results with the negative control to avoid mistaking non-specific amplification as desired bands.

## Method validation

We have optimized this direct PCR protocol to establish a method using minimal proteinase K incubation and inactivation times and have determined the optimal number of cells for use during PCR analysis. Jurkat and LCL cell lines were both used for various optimization experiments. To validate the Direct ENIT protocol, we tested several gRNAs in HeLa cells.

### Direct PCR protocol validation

We based our preliminary protocol design on previously reported direct-PCR protocols, one described by Ren and co-authors, and the second published online [3,4].

Suspension Jurkat cells were used to verify the applicability of direct PCR protocol for the performance of routine PCR reactions from cell cultures. Jurkat cells, either 5000, 25,000, or 100,000 cells, were pelleted at 1000 rcf for 2 min, in 200-μl PCR tubes. The supernatant was aspirated, and 2 × DreamTaq^TM^ buffer, 0.1 mg/ml proteinase K, and deionized water were added to the cell pellet to obtain a final volume of 10 µl. Resuspended cell pellets were incubated at 60 °C, for 1 h, and then proteinase K was inactivated, at 95 °C for 15 min. For PCR reactions, 1 µl cell suspension was used in PCR with DreamTaq^TM^ DNA Polymerase and the primers IGH_F/IGH_R (see Supplementary Table S1A). Samples that did not undergo proteinase K digestion were used for the negative control PCR.

**Fig. 2:**
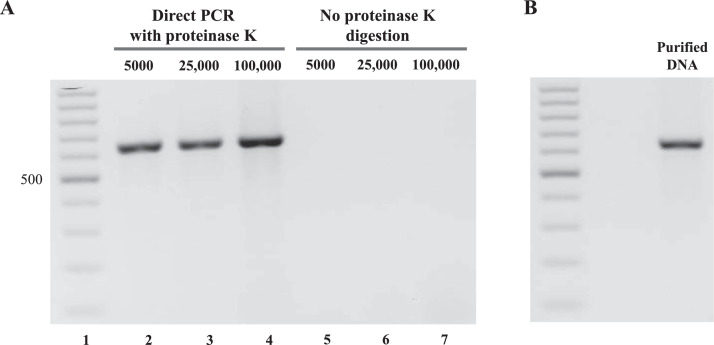
Direct PCR protocol validation. For direct PCR, a starting number of 5000 to 100,000 cells were used. The expected amplicon length was 644 bp. A. Lanes 2-4 show the direct PCR products from reactions using 5000, 25,000, and 100,000 Jurkat cells, whereas lanes 5-7 show the negative control PCR products obtained using the same cell numbers but without proteinase K digestion. В. Positive control shows the PCR performed using 60 ng purified genomic DNA.

After 40 cycles of PCR (PCR program 1, see Supplementary Table S2), we detected the presence of amplicons at the expected size, using agarose gel electrophoresis (Fig. 2). PCR was successful regardless of the starting cell number. No PCR product was detected in samples that did not undergo proteinase K digestion. The size and the amounts of direct PCR products were comparable to PCR with purification step.

### Optimization of proteinase K digestion time

**Fig. 3:**
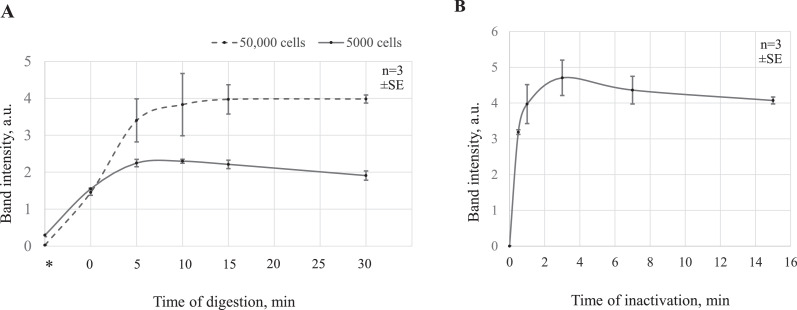
Optimization of proteinase K digestion and inactivation times. A*.* Direct PCR reactions using 5000 and 50,000 Jurkat cells (solid and dashed lines, respectively), with various proteinase K incubation times, were performed and analyzed by agarose gel electrophoresis. Each point represents the mean band intensity value of triplicate experiments, in arbitrary units. The error bars indicate the standard error (SE). Asterisks indicate direct PCR samples without proteinase K digestion. Gel images are shown in Supplementary Fig. S1. B. Band intensities on the agarose gel electrophoresis were analyzed for the direct PCR products of Jurkat cells, digested by proteinase K, using various heat inactivation times. Each point represents the mean band intensity value for three triplicate experiments (arbitrary units). The error bars indicate standard error (SE). Gel images are shown in Supplementary Fig. S2.

We performed a series of experiments to establish the minimal proteinase K digestion time necessary to achieve a successful PCR amplification. A starting number of 5000 or 50,000 Jurkat cells were pelleted by centrifugation at 800 rcf for 5 min. Pellets were resuspended in 0.1 mg/ml proteinase K and 2 × DreamTaq^TM^ Buffer solution and separated into five 10-μl reactions. Cell samples were incubated at 60 °C, for 0, 5, 10, 15, and 30 min. After proteinase K was heat-inactivated, at 95°C for 7 min, the full volume of the sample was used in a PCR reaction containing IGH_F/IGH_R primers and DreamTaq^TM^ DNA Polymerase. After 28 rounds of PCR (Program 1, Supplementary Table S2) the samples were analyzed by agarose gel electrophoresis, and the amount of product was estimated by quantifying the band intensity, using Fiji software [5] (Fig. 3A).

The quantification of PCR of samples following various proteinase K incubation times suggested that 10–15 min of proteinase K digestion was sufficient to provide the maximal amplification yield. These results also indicated that the reactions containing fewer cells required shorter incubation times. Even when proteinase K was added without any incubation time (point 0 on Fig. 3A time scale), a PCR product was detected, likely due to proteinase K activity during the gradient heating period of the inactivation reaction. A complete lack of proteinase K digestion resulted in no PCR product being detected (point * on Fig. 3A time scale).

### Optimization of proteinase K inactivation time

Next, we investigated whether the duration of the proteinase K inactivation period affects the amplification efficiency. A starting number of 50,000 Jurkat cells were pelleted and digested with 0.1 mg/ml proteinase K solution, in 2 × DreamTaq^TM^ Buffer, followed by 15 min incubation at 60 °C. Digestion was stopped by heating at 95 °C, for 0.5, 1, 3, 7, and 15 min, and the control sample was not inactivated. The full volume of the sample was used in a PCR reaction with DreamTaq^TM^ DNA Polymerase and IGH_F/IGH_R primers. The thermocycling conditions are described in Program 1, Supplementary Table S2. PCR products, after 28 amplification cycles, were separated by agarose gel electrophoresis, and the band intensities were quantified to estimate DNA amounts, using Fiji software (Fig. 3B). The results indicated that 3 min of proteinase K inactivation was sufficient for maximal PCR yield. As expected, no PCR product was detected in samples without prior proteinase K inactivation, as DNA polymerase is digested by active proteinase K.

### Evaluation of optimal cell number for direct PCR assay

To determine the optimal cell number for each direct PCR reaction, we examined PCR reactions using a series of cell dilutions (ranging from 160 to 500,000 Jurkat cells). Samples were pre-treated with 15 min proteinase K digestion and 3 min of proteinase inactivation. PCR was performed using DreamTaq^TM^ DNA Polymerase, as described above (28 rounds, Program 1, Supplementary Table S2).

The direct PCR products were analyzed by agarose gel electrophoresis, and the band intensities were quantified using Fiji software.

**Fig. 4:**
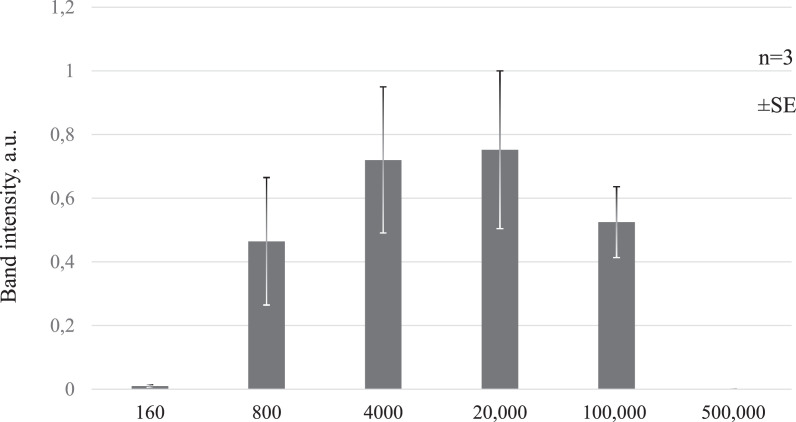
Estimation of the optimal number of cells in each reaction. Direct PCR results for varying numbers of Jurkat cells analyzed by agarose gel electrophoresis. Each bar represents the mean quantified band intensity for 3 experiments (arbitrary units). Gel images are shown in Supplementary Fig. S3. The error bars indicate standard error (SE).

The results indicated that 800–20,000 cells per reaction represented the optimal range for the successful performance of direct PCR. Fewer cell numbers did not provide sufficient PCR yields, and excessive cell lysate, containing 500,000 cells, appeared to inhibit the reaction (Fig. 4).

### The sensitivity of direct nested PCR

Using nested PCR in the ENIT approach is recommended, due to the higher sensitivity and specificity of nested PCR relative to conventional one-step PCR (see Supplementary Fig. S4). To estimate the sensitivity of the direct nested PCR approach (a detectable threshold of mutation-containing cells), we performed experiments using a mixed population of wild-type cells and cells with defined genotype.

Transgenic LCL cells, containing an integrated tetracycline activator gene (TA), were serially diluted in WT LCL cells in suspension (1:10, 1:100, 1:1000, 1:10,000, and 1:100,000). Then, 10,000 cells from each dilution sample were used for direct nested PCR. The first PCR round, using the outer primers TA_F_out/TA_R_out, was performed using DreamTaq^TM^ DNA Polymerase, according to the previously described protocol. The thermocycling conditions are described in Supplementary Table S2 (Program NEST1). An amount equal to 1/200 of the first-round PCR product was used in the second round of the nested PCR (Program NEST2, Table S2), using the inner primers TA_F_in/TA_R_in, as described in the Protocol section. We also used the IGH_F/IGH_R primers for positive amplification control. For primer sequences, see Supplementary Table S1A.

**Fig. 5:**
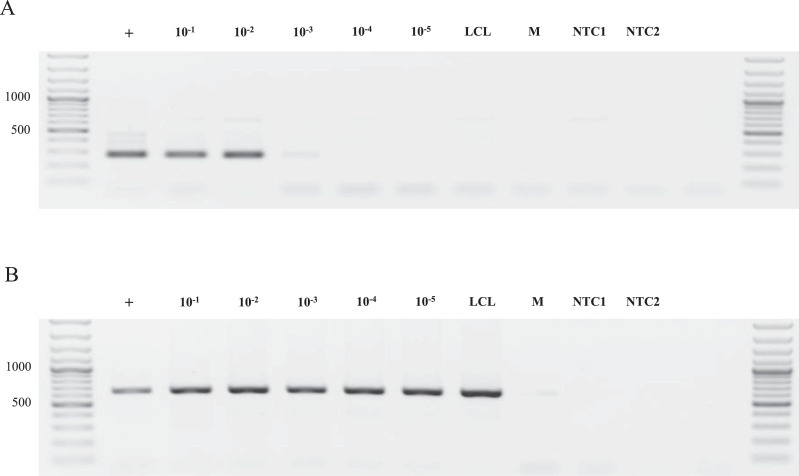
The sensitivity of direct nested PCR. LCL cells, encoding the TA transgene, were serially diluted in a WT LCL cell suspension (10^−1^–10^−5^). Direct nested PCR was performed on 10,000 cells from each mixture and analyzed by agarose gel electrophoresis. *A.* Direct nested PCR sensitivity testing (one representative experiment is shown). The expected amplicon length was 275 bp. *B.* Positive control for direct nested PCR. The expected amplicon length was 644 bp. «+» indicates samples with non-diluted transgenic cells; M — negative control medium sample; NTC1 — no template control for the first round of PCR; NTC2 — no template control for the second round of PCR.

PCR products were analyzed by agarose gel electrophoresis (Fig. 5). The results indicated that nested PCR for TA integration was positive at a minimal transgenic cell dilution of 10^−2^, indicating that 100 cells containing the transgene can be detected in a general population of 10,000 cells. A weak positive signal also was obtained in the sample with the transgenic cell dilution of 10^−3^. These results illustrated that the sensitivity threshold of direct nested PCR was approximately 1% of cells of the desired genotype in a general population.

### An example of direct ENIT application for gRNA testing

To validate the Direct ENIT approach, we assessed the performance of three gRNAs: gRNA_MLL, which targets a sequence in *MLL,* and gRNA_ARH1 and gRNA_ARH2, which target sequences in *ARHGAP26*. We used the pre-validated gRNA_MYC, which targets the *MYC* gene, and were expecting to detect *MYC-MLL* and *MYC-ARHGAP26* rearrangements. The target sequences of all gRNAs are provided in Supplementary Table S3.

HeLa cells were co-transfected with three plasmids, encoding Cas9, gRNA_MYC, and one of the tested gRNAs. 48 h after transfection, the direct nested PCR was performed, as described in the Protocol section. The outer and inner primers were designed to anneal to loci targeted by gRNA_MYC, gRNA_MLL, gRNA_ARH1, and gRNA_ARH2, as listed in Supplementary Table S1B. Positive control PCR was performed using MYC primers (Table S1B). The thermocycling conditions of the first and second rounds of nested PCR (ENIT1, 20 cycles and ENIT2, 30 cycles) are shown in Supplementary Table S2.

**Fig. 6:**
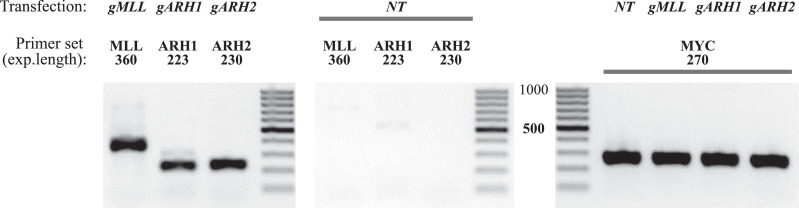
gRNA efficacy testing using the Direct ENIT approach. HeLa cells were co-transfected with Cas9 plasmid and two plasmids encoding gRNAs, the pre-validated gRNA_MYC, and one of the following gRNAs: gRNA_MLL (*gMLL*), gRNA_ARH1 (*gARH1*), or gRNA_ARH2 (*gARH2*). NT — non-transfected cells. The Direct ENIT approach was used to assess the efficacy of tested gRNAs, and the products of the second round of nested PCR were analyzed by agarose gel electrophoresis. Expected amplicon lengths are noted. Nested PCR primer sets are shown in Supplementary Table S1B.

The second-round PCR products were analyzed by agarose gel electrophoresis (Fig. 6). The specific amplification product of the translocation region was detected, indicating the successful gRNA targeting and CRISPR-mediated DSB induction. A negative control PCR performed on non-transfected cells did not result in the generation of a translocation product. All three tested gRNAs appeared to induce CRISPR/Cas9-mediated *MYC-MLL* and *MYC-ARHGAP26* translocations, which demonstrating their efficacy.

## Conclusion

To perform the easy and reliable gRNA efficacy validation in CRISPR/Cas9 experiments, we proposed a modification of the ENIT approach, using direct PCR. This modification results in a less time- and resource-consuming approach because it does not require genomic DNA isolation and purification steps and requires fewer cells per reaction. The direct, nested PCR strategy improves the sensitivity of the ENIT approach, allowing the detection of as few as 100 cells with induced translocation within a general population of 10,000 cells (compared with 700 cells with translocation among a general population of 7000 cells, in the original protocol [1]). The attained sensitivity was comparable to those of other assays used for gRNA efficacy testing, including T7-assay (0.5%) and SURVEYOR (3%) [6]. Moreover, the Direct ENIT approach is versatile and can be used to assess the performance of other gene-editing systems, based on targeted engineered nucleases [e.g., zinc finger nucleases (ZFNs) or transcription activator-like effector nucleases (TALENs)]. We optimized a direct PCR assay for use as a very fast alternative to conventional PCR, which requires a genomic DNA purification step.

## Declaration of Competing Interest

The authors declare that they have no known competing financial interests or personal relationships that could have appeared to influence the work reported in this paper.

## Appendix Supplementary materials

Image, application 1
