# Supplementary material for: Direct ENIT: An easy and reliable tool for gRNA efficacy verification by tracking induced chromosomal translocation
Source: MethodsX. 2020 Oct 16;7:101104. doi: 10.1016/j.mex.2020.101104 (PMC7588861; doi:10.1016/j.mex.2020.101104)
Supplement: Supplementary file 1 [file mmc1.docx]

## Supplementary material and Additional information:

### Background

CRISPR/Cas is a breakthrough gene-editing technology, which allows the genomes of cells and organisms to be altered for research and clinical purposes. The CRISPR/Cas system depends on the effector Cas endonuclease (Cas9, typically), which forms a complex with gRNA, a short hairpin RNA molecule that can recognize specific DNA sequence and “guide” the Cas endonuclease, to introduce a precise DSB in the target DNA. Although many bioinformatics tools exist for the prediction of gRNA efficiency [7], existing tools cannot assure sufficient cleavage efficacy during experiments. Inefficient editing complicates the detection and selection of cell clones containing desired mutations; therefore, determining the targeting efficacy of gRNAs should be performed before setting up a long-lasting experiment of genome editing.

Currently, many experimental tools exist for gRNA efficacy evaluation. The most common methods are the T7E1 and SURVEYOR endonuclease assays, which are based on the specific digestion of heteroduplexes, induced by the non-homologous end-joining (NHEJ)-scarring that accompanies CRISPR-mediated DSB induction. However, the sensitivities of these assays are limited by clonal diversity, and they require additional digestion steps. Next-generation sequencing, for the efficacy validation of targeted endonuclease-based systems, is considered to be the most comprehensive and reliable tool but is also the most expensive. The ENIT approach is based on induced translocations between endonuclease-targeted loci. These translocations belong to CRISPR-mediated mutations – less frequent then indels, but still occurring constantly.– The ENIT has been shown to be reliable and easy, requiring no specific reagents or equipment. However, this approach can only establish the relative efficacy of editing. Nevertheless, this approach is perfectly suited for the routine validation of gene-editing system efficacy. For a detailed review of the assays used for the efficacy evaluation of targeted endonuclease-based systems, see [8], [9].

[7] M. Haeussler, K. Schönig, H. Eckert, A. Eschstruth, J. Mianné, J.-B. Renaud, S. Schneider-Maunoury, A. Shkumatava, L. Teboul, J. Kent, J.-S. Joly, J.-P. Concordet

**Evaluation of off-target and on-target scoring algorithms and integration into the guide RNA selection tool CRISPOR**

Genome Biol., 17 (2016), p. 148. <https://doi.org/10.1186/s13059-016-1012-2>.

[8] N.A. Lomov, V.S. Viushkov, A.P. Petrenko, M.S. Syrkina, M.A. Rubtsov

**Methods of Evaluating the Efficiency of CRISPR/Cas Genome Editing**

Mol. Biol., 53 (6) (2019), pp. 862–875. <https://doi.org/10.1134/S0026893319060116>.

[9] D. Germini, T. Tsfasman, V. V Zakharova, N. Sjakste, M. Lipinski, Y. Vassetzky

**A Comparison of Techniques to Evaluate the Effectiveness of Genome Editing**

Trends Biotechnol., 36 (2) (2018), pp. 147–159. <https://doi.org/10.1016/j.tibtech.2017.10.008>.

### SUPPLEMENTARY

### Fig. S1. Various incubation time with proteinase K


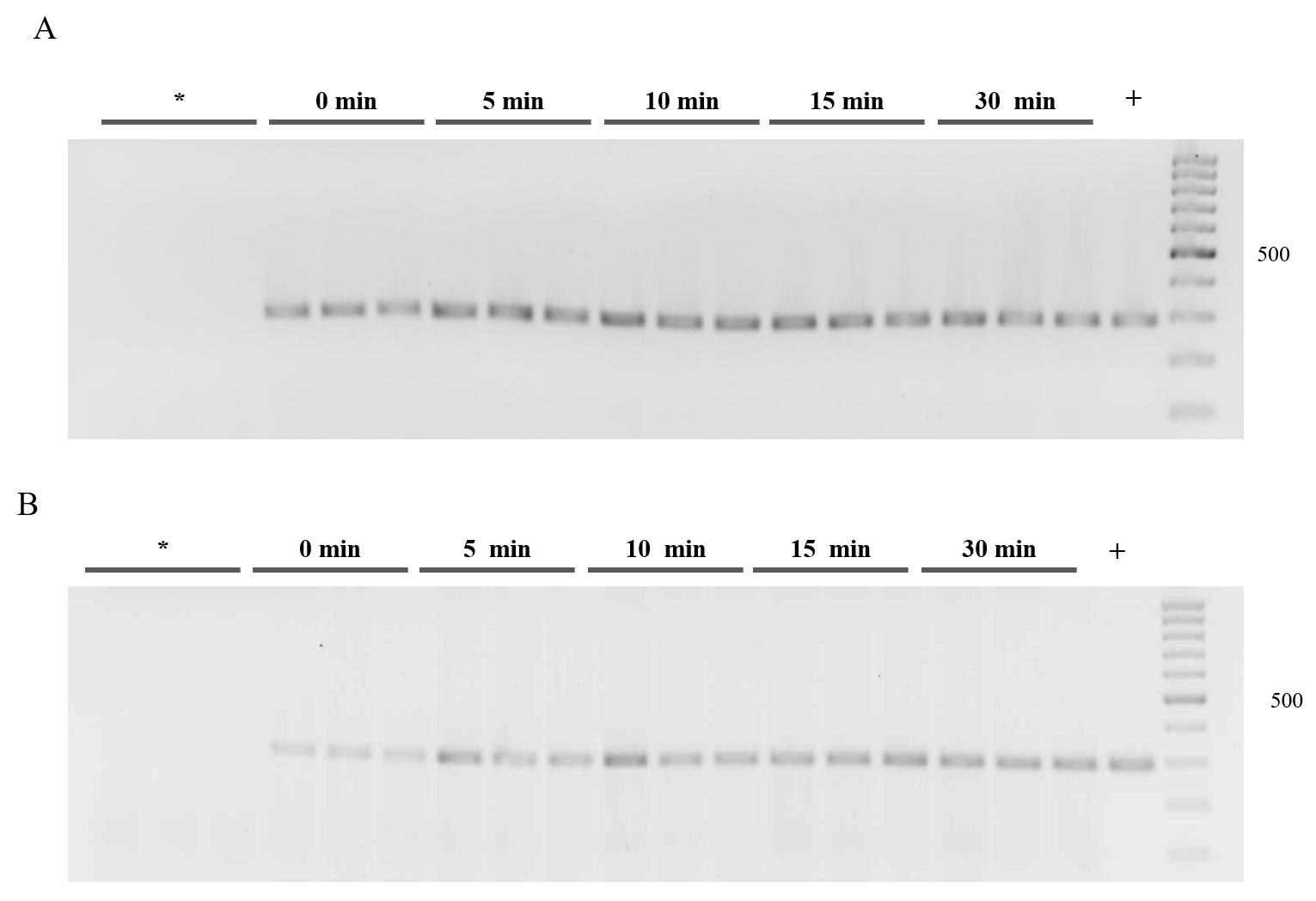


**Fig. S1. Various incubation time with proteinase K**. Direct PCR of cell samples incubated at 60°C with various proteinase K incubation times. Each direct PCR was performed in triplicate. MYC_F/MYC_R primers were used in PCR, expected amplicon length is 290 bp. Positive control (+) shows the PCR performed using of 60 ng purified genomic DNA. (*) indicates direct PCR samples without proteinase K digestion. ***A*** — 5,000 Jurkat cells per reaction, ***B*** — 50,000 Jurkat cells per reaction.

### Fig. S2. Various proteinase K inactivation time


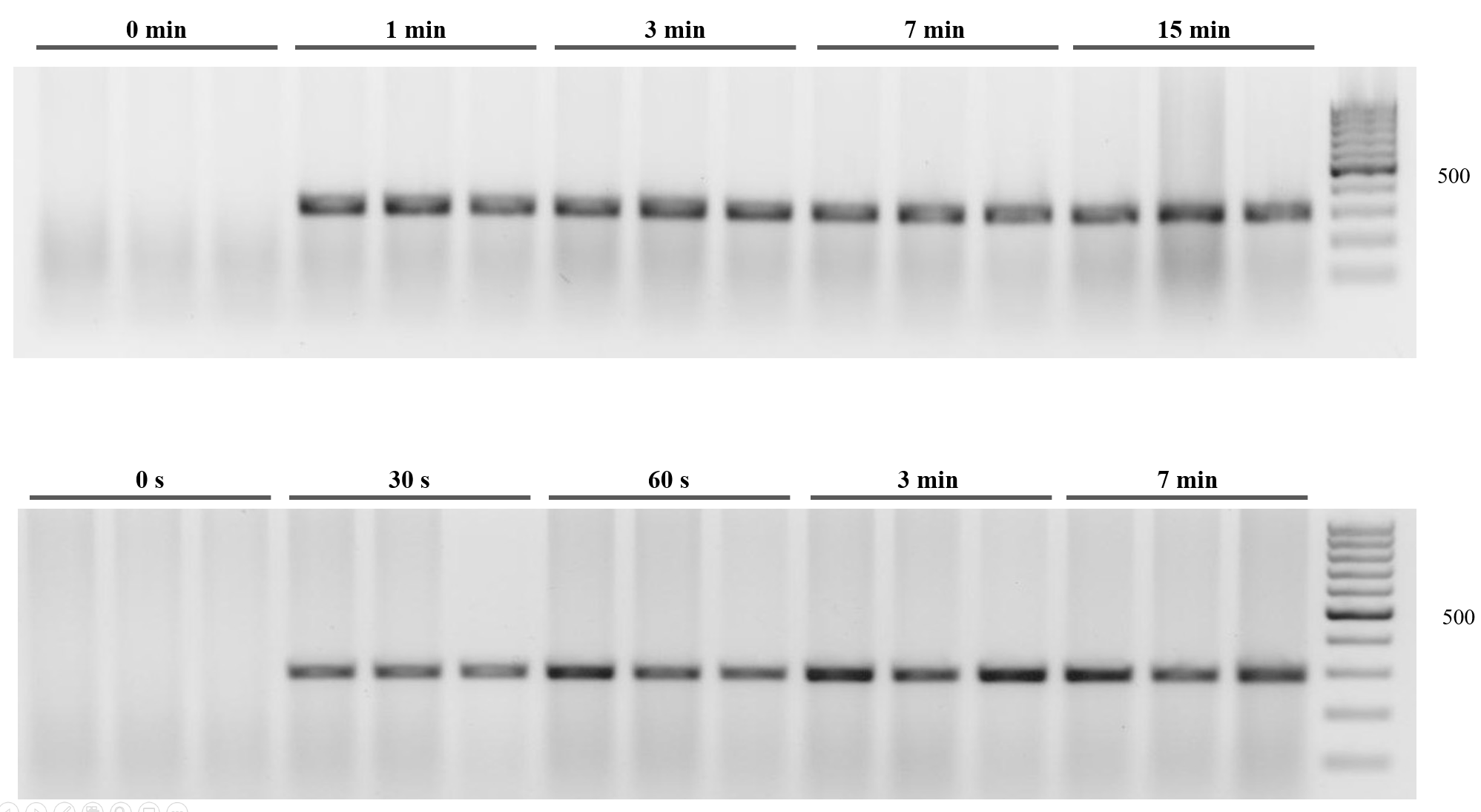


**Fig. S2. Various proteinase K inactivation time.** Jurkat cell samples were digested with proteinase K at 60°C for 15 min with following heat inactivation at 95°C for various times. Products of the following direct PCR with MYC_F/MYC_R primers were analyzed by gel electrophoresis. Expected amplicon length is 290 bp. Each direct PCR was performed in triplicate.

### Fig. S3. Evaluating the optimal cell number per direct PCR reaction


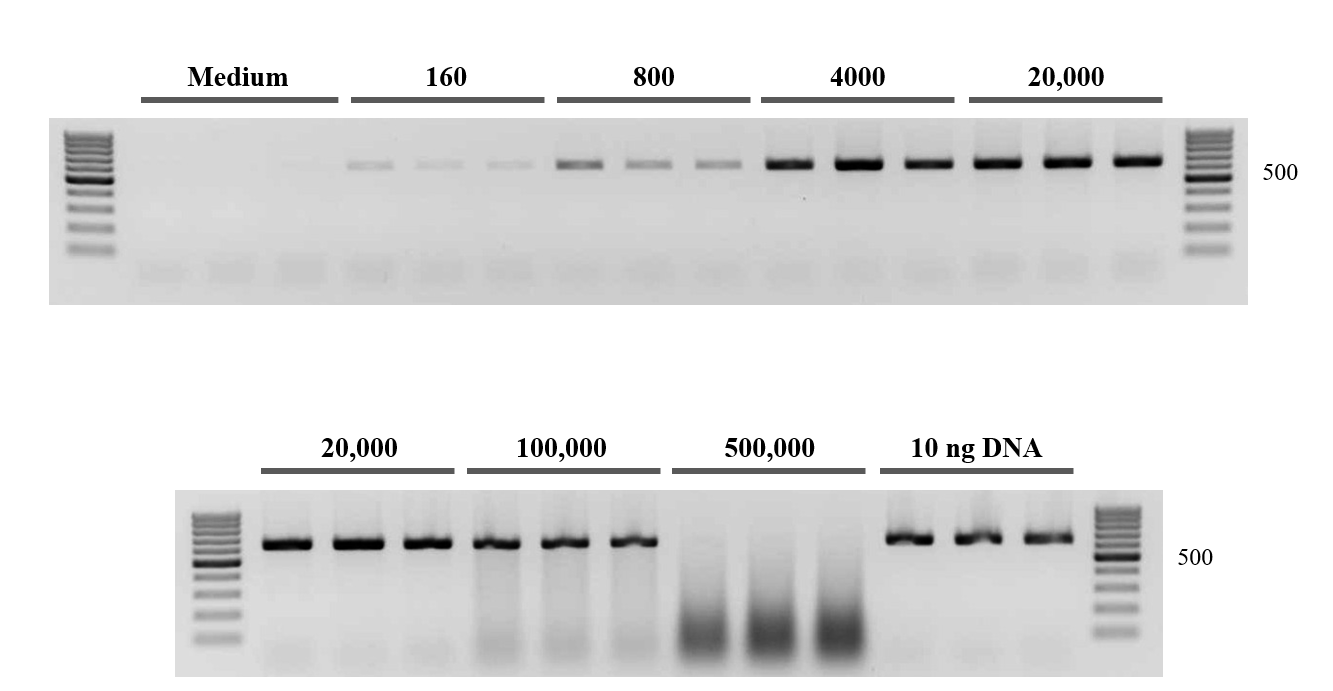


**Fig. S3. Evaluating the optimal cell number for direct PCR assay**. Varying numbers of proteinase K digested Jurkat cells (ranging from approximately 160 to 500,000 Jurkat cells, obtained by the serial dilution) were used in direct PCR with IGH_F/IGH_R primers (expected amplicon length is 644 bp). Each direct PCR was performed in triplicate. Positive control shows the PCR performed using of 10 ng purified genomic DNA. Negative control (Medium) shows the PCR performed with pure cell medium.

### Fig. S4. Comparison between one-step PCR and nested PCR in ENIT assay


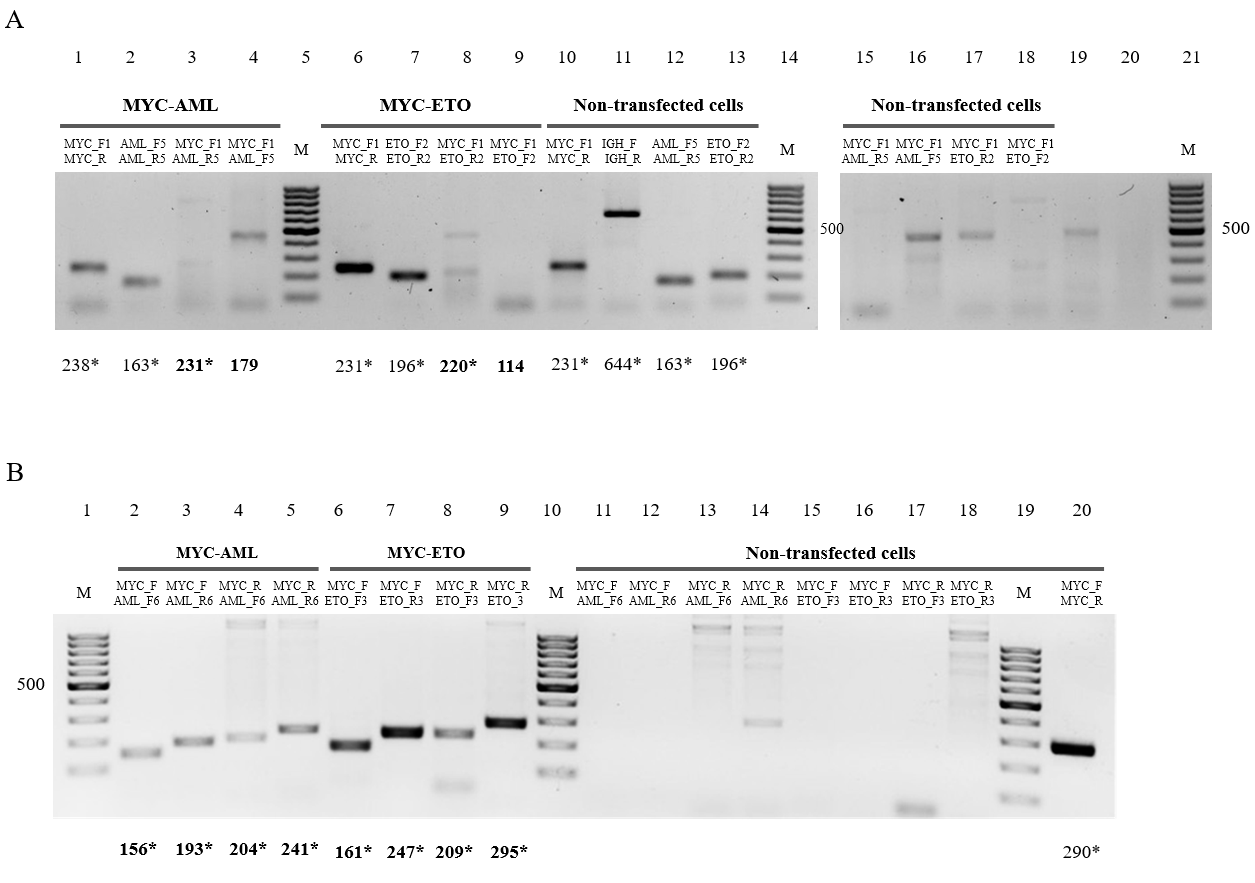


**Fig. S4. Comparison between one-step PCR (*A*) and nested PCR (*B*) in the ENIT assay**. In this experiment, the performance of two gRNA was tested in HeLa cells: gRNA_AML and gRNA_ETO, which target *AML* and *ETO*, respectively. TALEN, targeting *MYC,* was used to induce the second chromosome DSB, in each case (see Table S3 for target sequences). Non-transfected cells were used as a negative control. Primer pairs used for single PCR (***A***) or the second step of the two-step PCR (***B***) are represented above each lane, and their sequences are listed in Tables S1C and S1D, respectively. The expected amplicon sizes are mentioned below each lane. Asterisks mark PCR products that represent amplicons with the expected lengths.

When one-step PCR was used, some PCR products in the translocation region (lanes 4 and 9) were not detected, and the concentrations of others were low (lanes 3 and 8). Two-step PCR is a more reliable strategy, as each gRNA that was tested produced four possible translocation variants that could be detected (lanes 2-9). This experiment was performed using purified genomic DNA as a PCR template. Lanes 19 and 20 are not related to this experiment. PCR thermocycling conditions are shown in Table S2 (*Single* program for one-step PCR and *N1*, *N2* for two steps of nested PCR).

### Table S1A. Primers used for Direct ENIT optimization

| **Primer name** | **Sequences** | **Description** |
| --- | --- | --- |
| IGH_F | CCCAGCCCTTGTTAATGGACT | IGH_F/ IGH_R primer pair amplifying 644 bp fragment of Immunoglobulin heavy chain locus (*IGH*). |
| IGH_R | AGGTCCCCTTGCTCTAGAAGT |  |
| TA_F_out | TGCCAATCGAGATGCTGGAC | TA_F_out/ TA_R_out outer primer pair for TA transgene detection in LCL cells (648 bp) |
| TA_R_out | GCATTCTAGTTGTGGTTTGTCCA |  |
| TA_F_in | AAATCAGCTCGCGTTCCTGT | TA_F_in/ TA_R_in inner primer pair for TA transgene detection in LCL cells (275 bp) |
| TA_R_in | CGCTTTCGCACTTTAGCTGT |  |
| MYC_F | CCAGTAACTCCTCTTTCTTCGGAC | MYC_F/ MYC_R primer pair amplifying 290 bp fragment of *MYC* gene |
| MYC_R | CGCTATGCTGGATTTTGCTGCA |  |

### Table S1B. Primers used for Direct ENIT validation

| **Primer name** | **Sequences** | **Description** |
| --- | --- | --- |
| MYC_F2_out | AAATGCTGGCTTTGCCAAACT | Basic outer forward primer for Direct ENIT validation (nested PCR first round) |
| MLL_R_out | TGCCATTGGAGAGAGTGCTG | Outer primer for Direct ENIT validation of gRNA_MLL (nested PCR first round) |
| ARH1_R_out | AAATAGCCACCTGCCACCAA | Outer primer for Direct ENIT validation of gRNA_ARH1 (nested PCR first round) |
| ARH2_R_out | CATCCACCAGGTGACAACGA | Outer primer for Direct ENIT validation of gRNA_ARH2 (nested PCR first round) |
| MYC_F2_in | AGGGAAACAAGTGTTCATGGGA | Basic inner primer for Direct ENIT validation (nested PCR second round) |
| MLL_R_in | GACCGGAGGTGGTTTTTCCT | Inner primer for Direct ENIT validation of gRNA_MLL (nested PCR second round) |
| ARH1_R_in | TCCTCAAGCCTATGGGGTACT | Inner primer for Direct ENIT validation of gRNA_ARH1 (nested PCR second round) |
| ARH2_R_in | GAGGCATCTCAAGACTAGGCA | Inner primer for Direct ENIT validation of gRNA_ARH2 (nested PCR second round) |
| MYC_R2 | CAGCTGGCTGTTGTCGGGTC | Basic reverse primer for Direct ENIT validation (nested PCR both first and second rounds) |

### Table S1C. Primers used for one-step PCR ENIT (Fig. S4A)

| **Primer name** | **Sequence** |
| --- | --- |
| MYC_F1 | AGGAGGTGGCTGGAAACTTGT |
| MYC_R1 | CGCTATGCTGGATTTTGCTGCA |
| AML_F5 | CAGGCTTTTCACAAGCATTCCT |
| AML_R5 | TGGGGAAGCTCACCAGATAG |
| ETO_F2 | TGCATGAACATAAACAGGCACTC |
| ETO_R2 | TTGGGACACCTAGGAGTGGT |
| IGH_F | CCCAGCCCTTGTTAATGGACT |
| IGH_R | AGGTCCCCTTGCTCTAGAAGT |

### Table S1D. Primers used for two-step PCR ENIT (Fig. S4B)

| **Primers used for first PCR (outer primers)** | | **Primers used for second PCR (inner primers)** | |
| --- | --- | --- | --- |
| Primer name | Sequence | Primer name | Sequence |
| MYC_F2 | CCAGTAACTCCTCTTTCTTCGGAC | MYC_F2 | CCAGTAACTCCTCTTTCTTCGGAC |
| MYC_R1 | CGCTATGCTGGATTTTGCTGCA | MYC_R1 | CGCTATGCTGGATTTTGCTGCA |
| AML_F5 | CAGGCTTTTCACAAGCATTCCT | AML_F6 | TCCTTCTTCCCTGAAGCCGA |
| AML_R5 | TGGGGAAGCTCACCAGATAG | AML_R6 | CTCCAGTGGGCCTATTCTGC |
| ETO_F2 | TGCATGAACATAAACAGGCACTC | ETO_F3 | GAACATAAACAGGCACTCTTTGC |
| ETO_R2 | TTGGGACACCTAGGAGTGGT | ETO_R3 | GGCCTGAGGAGCTTGCTATC |

### Table S2. PCR Protocols

| **Protocol name** | **Initial denaturation and polymerase activation** | **PCR cycle** | | | **Number of cycles** | **Final extension** |
| --- | --- | --- | --- | --- | --- | --- |
|  |  | Denaturation | Annealing | Extension |  |  |
| 1 | 95 °С 3 min | 95 °С 15 s | 61 °C 30 s | 72 °C 1 min | 40 (28) | 72 °C 10 min |
| NEST1 | 95 °С 7 min | 95 °С 30 s | 59 °C 1 min | 72 °C 30 s | 20 | 72 °C 5 min |
| NEST2 | 95 °С 3 min | 95 °С 15 s | 59 °C 1 min | 72 °C 30 s | 25 (30) | 72 °C 5 min |
| ENIT1 | 95 °С 5 min | 95 °С 30 s | 58 °C 90 s | 72 °C 45 s | 20 | 72 °C 5 min |
| ENIT2 | 95 °С 5 min | 95 °С 10 s | 58 °C 1 min | 72 °C 30 s | 30 | 72 °C 5 min |
| Single | 95 °С 10 min | 95 °С 15 s | 60 °C 1 min | 72 °C 1 min | 40 | 72 °C 5 min |
| N1 | 95 °С 5 min | 95 °С 20 s | 60 °C 1 min | 72 °C 45 s | 20 | 72 °C 5 min |
| N2 | 95 °С 5 min | 95 °С 20 s | 60 °C 1 min | 72 °C 30 s | 25 | 72 °C 5 min |

### Table S3. Sequences recognized by subunits of TALE-nuclease and guide RNAs

| **Name** | **Recognized sequence** |
| --- | --- |
| TALEN MYC subunit 1 | TCCCCCGCTGGAAACCTTG |
| TALEN MYC subunit 2 | TGCTCCTGCCCCCACCTGA |
| gRNA AML | GACTCCCCCATGTACCCCTA |
| gRNA ETO | GCTTGGGAGGAAAGAATGAC |
| gRNA MYC | TATCCCTTAAAGCGCTGACG |
| gRNA_MLL | ATCACGAGCCCACAAGGTCT |
| gRNA_ARH1 | GCTCTGCCTAAATCGAATCC |
| gRNA_ARH2 | CACCAGCAAGAACGCTTCCG |
